# Electrical tuning effect for Schottky barrier and hot-electron harvest in a plasmonic Au/TiO_2_ nanostructure

**DOI:** 10.1038/s41598-020-79746-5

**Published:** 2021-01-11

**Authors:** Zhiguang Sun, Yurui Fang

**Affiliations:** Key Laboratory of Materials Modification By Laser, Electron, and Ion Beams (Ministry of Education), School of Physics, Dalian University of Technology, Dalian, 116024 People’s Republic of China

**Keywords:** Nanophotonics and plasmonics, Energy harvesting, Nanoparticles

## Abstract

Schottky barrier controls the transfer of hot carriers between contacted metal and semiconductor, and decides the performance of plasmonic metal–semiconductor devices in many applications. It is immensely valuable to actively tune the Schottky barrier. In this work, electrical tuning of Schottky barrier in an Au-nanodisk/TiO_2_-film structure was demonstrated using a simple three-electrode electrochemical cell. Photocurrents excited at different wavelength significantly increase as the applied bias voltage increases. Analyzing and fitting of experimental results indicate that the photocurrent is mainly affected by the bias tuning position of Schottky barrier maximum, which shifts to metal–semiconductor interface as applied voltage increases, and enhances the collection efficiency of the barrier for plasmonic hot electrons. The conduction band curvature of 0.13 eV was simultaneously obtained from the fitting. This work provides a new strategy for facile tuning of Schottky barrier and hot-electron transfer across the barrier.

## Introduction

Plasmonic nanostructures have greatly strong ability to harvest photon energy by free electrons collective oscillation under light excitation, which is known as localized surface plasmonic resonance (LSPR)^1^. Its excellent properties such as strong field confinement, largely enhanced light-matter interaction, make LSPR extensively employed for scientific researches and emerging applications of ultrasensing^2^, nanolasing^3^, waveguide^4^, photothermal therapies^5^, et al. When metal nanostructure is in contact with semiconductor, their different work functions induce a space-charge region with internal electric field, bending semiconductor bands, and give rise to a Schottky barrier on metal–semiconductor interface^6^. High energy hot carriers excited via surface plasmon non-radiative decay by Landau damping can overcome Schottky barrier and transfer from metallic nanostructures to the contacted semiconductor^7^. The Schottky barrier significantly inhibits hot-carrier recombination and facilitates hot-carrier separation, which plays a key role in photocatalytic reactions and photovoltaic devices^8,9^.

In plasmonic metal–semiconductor devices based on hot carriers, the transfer of hot carriers between contacted metal and semiconductor is one of the most important aspect that determines the device performance. Efforts have been made to control the hot-carrier transfer by different means. Shi et al. tuned Au–TiO_2_ interfacial structure by annealing at different temperatures. The closer contact between Au and TiO_2_ promoted hot-electron transfer across the interface^10^. Decorating metal or semiconductor was also investigated to control the hot-carrier transfer. The addition of decorating materials or functional groups provides new hot-carrier transfer pathways across the interface^11–13^. More works aim to control the hot-carrier transfer by tuning the Schottky barrier, which promotes the separation of hot carriers and also resists their directional migration. Diversely means were proposed to tune the Schottky barrier, such as changing carrier density by adjusting doping concentration and oxygen vacancies of metal oxide semiconductors^14–16^, modifying the metal–semiconductor interface by plasma treatment^17^, molecule capping^18^ and insertion layer^19,20^, electrostatic gating and applying strain on two-dimensional semiconductors^21–24^, and simply applying external bias voltage or gate light^25–29^. Among the strategies above, electrical tuning of Schottky barrier is quite promising. It achieves reversible and active tuning, but limited by the requirement of ingenious cell structure and precise operation for connecting nanostructures with electrodes. Facile and reversible methods still need developing.

On the other hand, most works of tuning Schottky barrier mainly focus on the change of Schottky barrier height and corresponding effects. In fact, the Schottky barrier is much more complex than the simple models frequently used^6^. As Schottky barrier height is tuned, other parameters of Schottky barrier may also change, such as width of depletion region, position of Schottky barrier maximum, height of barrier caused by band curvature, etc.^30^. These factors may seriously affect the transfer of hot carriers, but they were usually ignored.

In this work, Schottky barrier of an Au-nanodisk/TiO_2_-film (AT) structure was electrically tuned in a simple three-electrode electrochemical cell. As the increase of reverse bias voltage, photocurrent originated from plasmonic hot-electron transfer from Au nanodisks to TiO_2_ significantly increases. Based on fitting and analyzing of experimental results, it is found that the increase of photocurrent is mainly induced by electrical tuning of Schottky barrier maximum position, which improves collection efficiency of the barrier, rather than Schottky barrier height.

## Results and discussion

**Figure 1:**
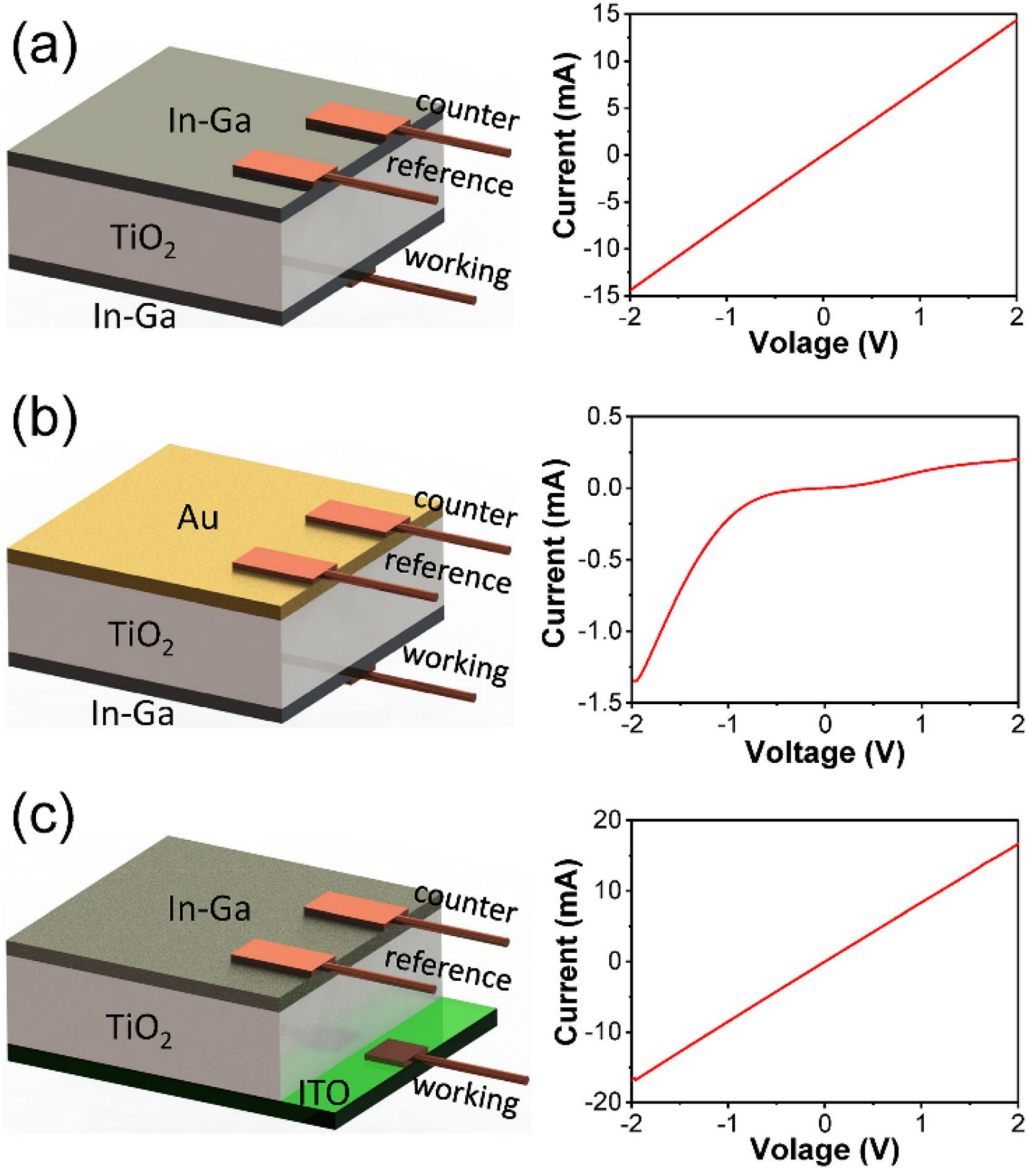
Schematic illustrations of (**a**) In-Ga/TiO_2_/In-Ga, (**b**) Au/TiO_2_/In-Ga and (**c**) In-Ga/TiO_2_/ITO cells (left), and their measured *I–V* curves (right).

To achieve the electrical tuning of Schottky barrier and further control of hot-electron injection from Au to TiO_2_, an external bias should be effectively applied on the Au–TiO_2_ Schottky junction. In this sense, the existence of Schottky barrier on Au–TiO_2_ interface of the investigated structure should be confirmed, while barrier on other interfaces in the structure should be avoided. In Fig. 1a, the current of an In-Ga/TiO_2_/In-Ga structure is proportional to voltage from − 2 to 2 V, showing an Ohmic characteristic. In-Ga alloy connects TiO_2_ and electrode with an Ohmic contact, so the contact properties of TiO_2_ with Au or ITO can be revealed by replacing one of the In-Ga alloy layers. In Au/TiO_2_/In-Ga structure, the Schottky contact of Au and TiO_2_ can be corroborated by the nonlinear (current–voltage) *I–V* curve in right panel of Fig. 1b. The Schottky barrier height calculated from *I–V* data by Cheung’s method is approximately 0.8 eV^31,32^. Whereas, for In-Ga/TiO_2_/ITO structure, the linear *I*–*V* plot in Fig. 1c indicates that ITO and TiO_2_ are also in Ohmic contact. Therefore, in AT structure fabricated on ITO glass, there is Schottky barrier only on the interface of Au and TiO_2_.

When AT structure was tested as working electrode in an electrochemical cell, voltage drop also occurs at metal-electrolyte interfaces, including those of nanoparticle-electrolyte and counter electrode–electrolyte, because of electric double layer on the interfaces. However, the existence of Au–TiO_2_ Schottky barrier makes this fraction prone to be small. Besides, the different Fermi levels of TiO_2_ and electrolyte give rise to a space charge layer at TiO_2_-electrolyte interface, and further induce another TiO_2_-electrolyte barrier on the interface. The barrier is parallel with Au–TiO_2_ Schottky barrier, avoiding short circuiting the latter. Therefore, external bias can be effectively applied on the Schottky junction of Au and TiO_2_, while a fraction of voltage drop inevitably exists on other interfaces and components of the investigated system. Electrical tuning of the Schottky barrier can be revealed by experiments at different bias voltages.

**Figure 2:**
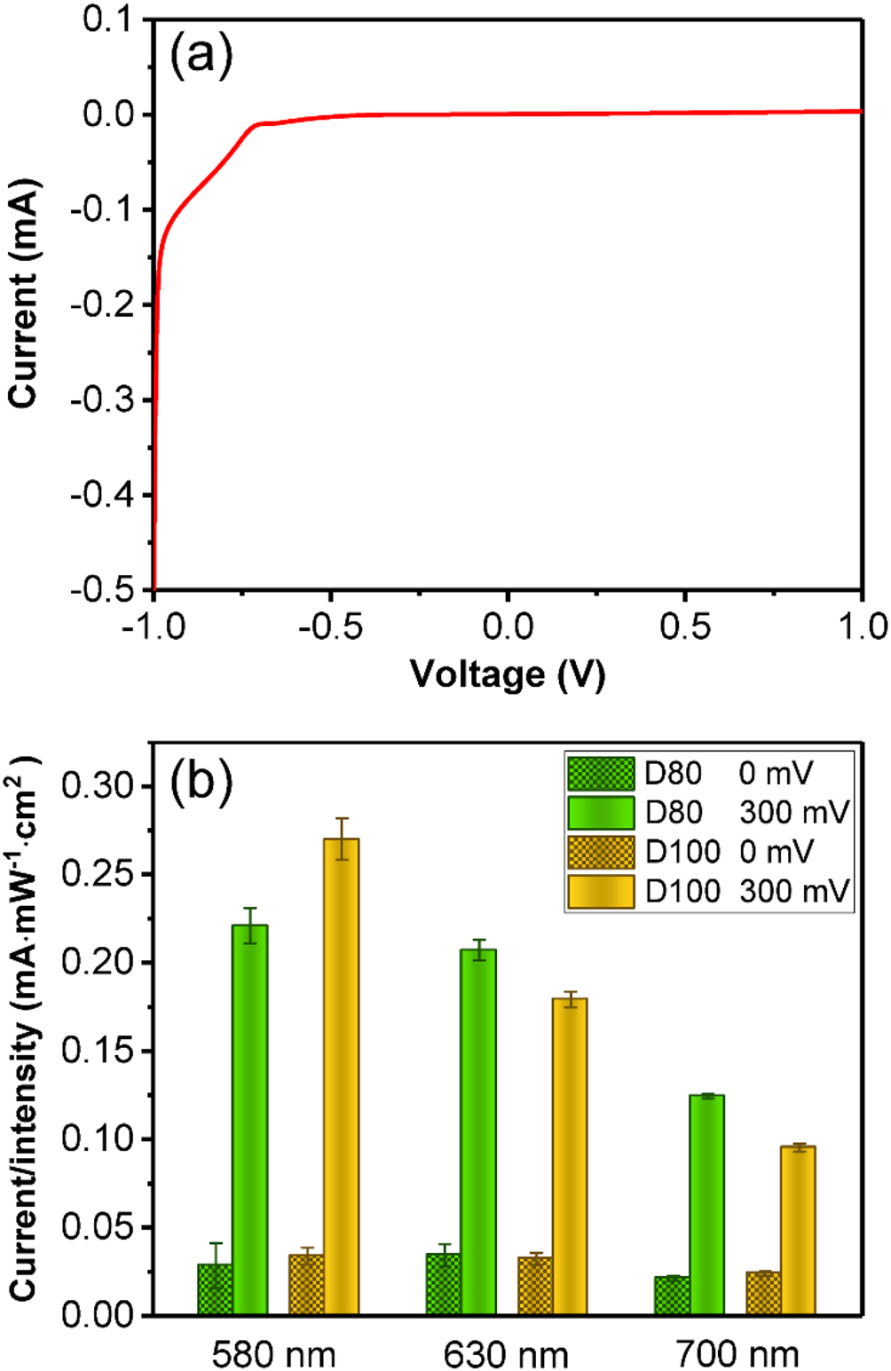
(**a**) *I–V* curve of AT with 80 nm nanodisk diameter. (**b**) Ratio of photocurrent and light intensity for AT samples with 80 nm and 100 nm nanodisk diameters at bias voltages of 0 mV and 300 mV, and at excitation wavelength of 580, 630 and 700 nm.

*I*–*V* curve of AT sample in the electrochemical cell was also measured and shown in Fig. 2a. The nonlinear curve and calculated Schottky barrier height of approximately 0.8 eV is in accordance with the analysis above. Figure 2b shows the photocurrents of AT structure with nanodisk diameters of 80 nm (D80) and 100 nm (D100) at different bias voltages. Excited by incident light, hot electrons generated from localized surface plasmon (LSP) decay in Au nanodisks pass through the Schottky barrier to the TiO_2_ film, and induce positive photocurrents. When 300 mV bias was applied, the photocurrents of both samples significantly increase several times. Considering hot electrons excited at a certain wavelength in nanodisks have almost the same energy distribution, it can be inferred that the enhancement of photocurrent comes from the electrical tuning effect for the Schottky barrier, which affects the hot-electron transfer efficiency from Au nanodisks to TiO_2_ film.

**Figure 3:**
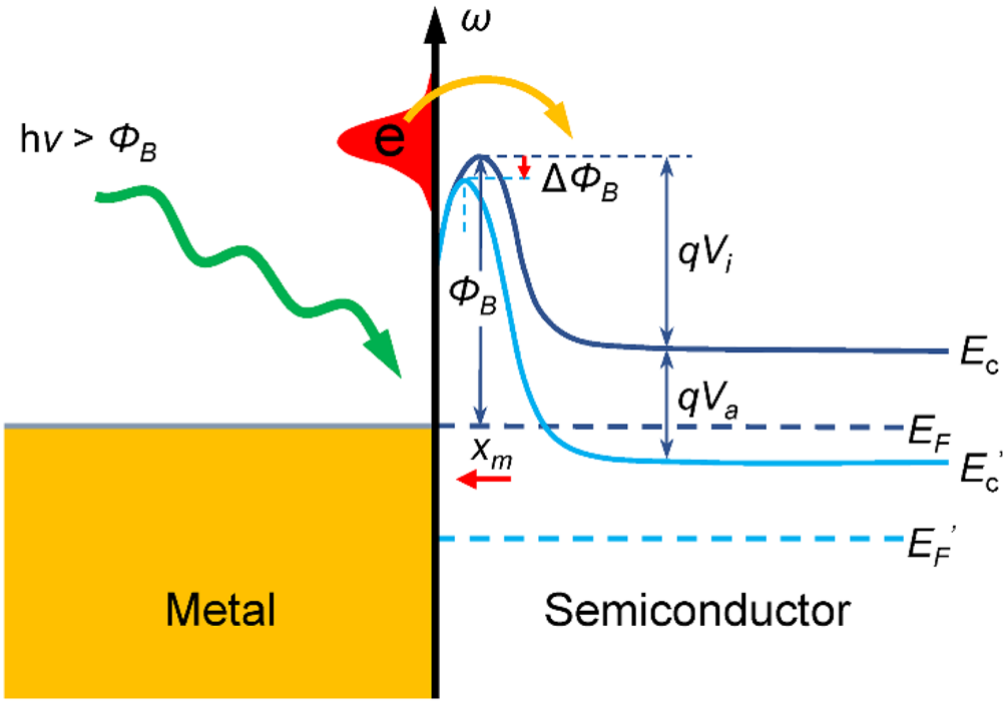
Schematic illustrations of hot-electron generation and energy bands for a Schottky junction without bias (dark blue lines) and under reverse bias (sky blue lines).

Bias tunes the Schottky barrier in different aspects as illustrated in Fig. 3. When a reverse bias ($V_{a} < 0$, positive on the semiconductor side, negative on the metal side) is applied to the Schottky junction, Fermi level of the semiconductor ($E_{f}$) decreases at a degree of $\left| {qV_{a} } \right|$ to $E_{f}^{^{\prime}}$. And semiconductor conduction band minimum also decreases from $E_{c}$ to $E_{c}^{^{\prime}}$, while the potential barrier resulted from conduction band curvature increases from $qV_{i}$ to $q\left( {V_{i} - V_{a} } \right)$ ^30^. Due to the effect of mirror charges induced in the metal by electrons in the semiconductor, the height of Schottky barrier ($\Phi_{b}$) is slightly affected by applied voltage and decreases at a degree of

1 $$ \Delta \Phi_{b} = \sqrt[4]{{\frac{{q^{3} N_{d} }}{{8\pi^{2} \varepsilon_{s}^{3} }}\left( {V_{i} - V_{a} } \right)}} $$

where *q*, $N_{d}$ and $\varepsilon_{s}$ are electron charge, donor carrier concentration of semiconductor and semiconductor permittivity, respectively^30^. The resulting Schottky barrier height equals $\Phi_{b} - \Delta \Phi_{b}$. Meanwhile, the Schottky barrier maximum position ($x_{m}$) is also affected by mirror charges and varies with applied voltage as^30^

2 $$ x_{m} = \frac{1}{4}\sqrt[4]{{\frac{q}{{2\pi^{2} \varepsilon_{s} N_{d} \left( {V_{i} - V_{a} } \right)}}}} $$

It can be seen that as the magnitude of applied reverse voltage increases, *x*_*m*_ decreases and Schottky barrier shifts close to the metal–semiconductor interface.

Incident light with photon energy higher than Schottky barrier excites hot electrons in the metal and transports them from metal to semiconductor conduction band. This effect is also called internal photoemission and its efficiency ($\eta$) is influenced by multiple factors as follows:

3 $$ \eta = AF_{e} P_{E} \eta_{c} $$

where $A$ is optical absorbance of the metal part, $F_{e}$ is the fraction of photons generating photoelectrons and contributing to photocurrent, $P_{E}$ is the probability of photoexcited electrons overcoming Schottky barrier after scattering with cold electrons and boundary surface, $\eta_{c}$ is collection efficiency of the barrier. Among the above four parameters, $A$ and $P_{E}$ are independent on Schottky barrier, while $F_{e}$ and $\eta_{c}$ are functions of Schottky barrier height and Schottky barrier maximum position, respectively^33^.

**Figure 4:**
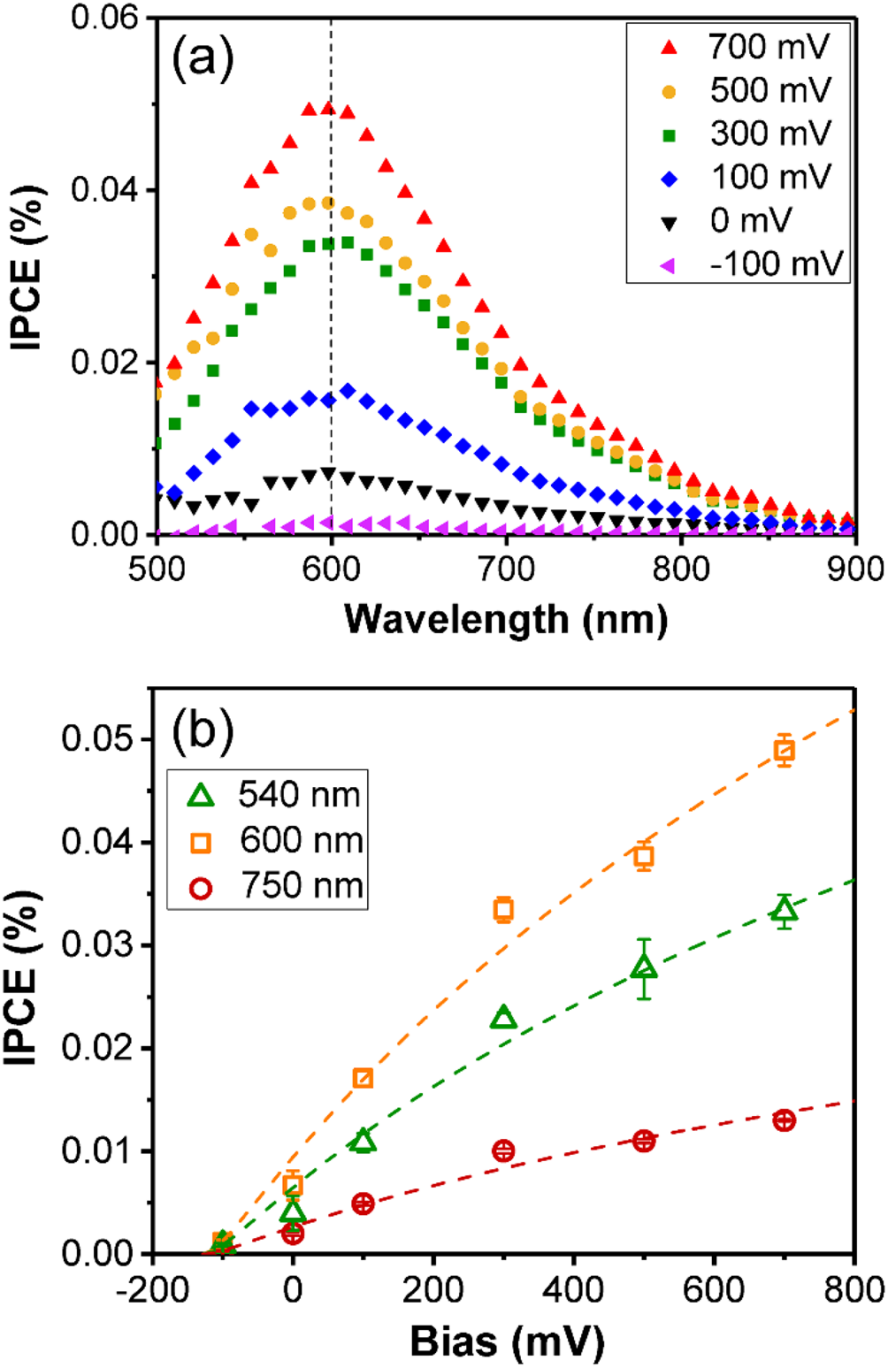
(**a**) IPCE data for AT sample of 80 nm nanodisk diameter at different bias voltages, and (**b**) variation of IPCEs with bias at 540, 600 and 750 nm wavelength from experimental data (hollow marks) and fitting results (dash curves).

To reveal how the bias tuning Schottky barrier affects internal photoemission and hot electron harvest, relations between bias and $F_{e}$, $\eta_{c}$ are analyzed. Incident photon-to-electron conversion efficiencies (IPCEs) of AT structure (D80) at different bias voltages were measured and calculated according to

4 $$ {\text{IPCE}} = \frac{{n_{e} }}{N} = \frac{h \cdot c \cdot I}{{e \cdot P \cdot \lambda }} $$

where *n*_*e*_, *N*, *h*, *c*, *I*, *e*, *P* and *λ* are collected photoelectron number, incident photon number, Plank constant, light velocity, photocurrent, electron charge, incident light power, and light wavelength, respectively. As Fig. 4a shows, IPCEs at all bias conditions exhibit a peak around 600 nm for LSPR of the structure. At this condition, the resonance results in an enhancement of plasmonic hot electron generation. Besides, the IPCEs significantly increase with the bias from − 100 to 700 mV, which is coherent with the photocurrents in Fig. 2. The peak IPCE with 700 mV bias is as high as about seven times of that without bias. Estimating from Eq. (2) with common TiO_2_ parameters, bias (< 1 V) induced Schottky barrier height variation for an Au–TiO_2_ interface is quite small (tens of milli-electron-volts)^29,34^. Compared with the total Schottky barrier height near 1 eV, the influence of applied voltage on $F_{e}$, which is a function of Schottky barrier height, can be neglected, and it cannot support the significant increase of IPCEs with bias.

Then, it comes to the relation between bias and $\eta_{c}$. The barrier collection efficiency $\eta_{c}$ indicates the probability of hot electrons migrating from metal interface to Schottky barrier maximum without scattering, and is given by

5 $$ \eta_{c} = e^{{ - \frac{{x_{m} }}{{L_{s} }}}} $$

where $L_{s}$ is electron scattering length in TiO_2_^33^. Combining Eqs. (2) and (5), it is known

6 $$ \eta_{c} = e^{{ - B\left( {V_{i} - V_{a} } \right)^{{ - \frac{1}{4}}} }} $$

where $$B$$ is $$\frac{1}{{4L_{s} }}\sqrt[4]{{\frac{q}{{2\pi^{2} \varepsilon_{s} N_{d} }}}}$$, independent of bias and Schottky barrier. In Fig. 4b, data points of IPCE with bias voltage at different excitation wavelength obtained from Fig. 4a were fitted using the relation of $${\text{IPCE}} = ke^{{ - B\left( {V_{i} - V_{a} } \right)^{{ - \frac{1}{4}}} }}$$. Fitting parameters were given in Table 1. According to Eq. (3), $$k$$ is proportional with $$AF_{e} P_{E}$$ and varies with $$ A$$ at different wavelength. It is clear that the fitted dash curves in the figure match the experimental data well. Moreover, fitting at different wavelength is in good agreement, having the same fitting parameters of $$V_{i} = 130{ }\;{\text{mV}}$$ and $$B = 15$$. The obtained conduction band curvature $$qV_{i}$$ of 0.13 eV here is quite close to the ones in references^35^, and it further supports the fitting results. Therefore, it can be deduced that the hot-electron harvest efficiency of the plasmonic Au/TiO_2_ structure is mainly determined by the bias tuning Schottky barrier maximum position $$x_{m}$$. Taking $$N_{d} = 2.5 \times 10^{25} {\text{m}}^{ - 3}$$, $$\varepsilon_{s} = 8.85 \times 10^{ - 10} \;{\text{F}} \cdot {\text{m}}^{ - 1}$$ from a reference with similar samples into Eq. (2), $$x_{m}$$ can be estimated to be 0.3 nm^36^. For $$x_{m}$$ is proportional to $$\left( {V_{i} - V_{a} } \right)^{{ - \frac{1}{4}}}$$, as the reverse bias increases, the Schottky barrier maximum position shifts to the metal–semiconductor interface and decreases to 63% of the primitive one at 700 mV reverse bias. In fact, $$x_{m}$$ can be further electrically tuned to 56% of the primitive value at a reverse bias voltage of 1.2 V, above which the electrolysis of water will happen. The deduced Schottky barrier maximum position significantly enhances the hot-electron collection efficiency of the Schottky barrier, and thus achieves an improved photocurrent.

**Table 1:** Fitting parameters of IPCEs varying with bias at different wavelength.

*λ* (nm)	*k*	*B*	*V*_*i*_ (mV)
540	0.55	15	130
600	0.80	15	130
750	0.23	15	130

## Conclusion

In conclusion, Schottky barrier of AT structure was electrically tuned in a simple three-electrode electrochemical cell. Appling a reverse bias, photocurrents and IPCEs at different excitation wavelength significantly increase as the applied voltage increases. According to fitting results, the photoelectric response obeys a relation of ${\text{IPCE}} = ke^{{ - B\left( {V_{i} - V_{a} } \right)^{{ - \frac{1}{4}}} }}$. It indicates the photocurrent is mainly controlled by bias tuning Schottky barrier maximum position, rather than Schottky barrier height. Schottky barrier maximum position increases photocurrent via shifting to the interface and enhancing collection efficiency of Schottky barrier for hot electrons. The conduction band curvature of Schottky barrier was also obtained to be 0.13 eV from the fitting. This work suggests a new strategy to facilely and reversely tune the Schottky barrier and hot-carrier transfer across the barrier. It is highly beneficial to improve the performance of plasmonic hot-carrier devices in photocatalysis and photovoltaic systems.

## Methods

### Sample fabrication

**Figure 5:**
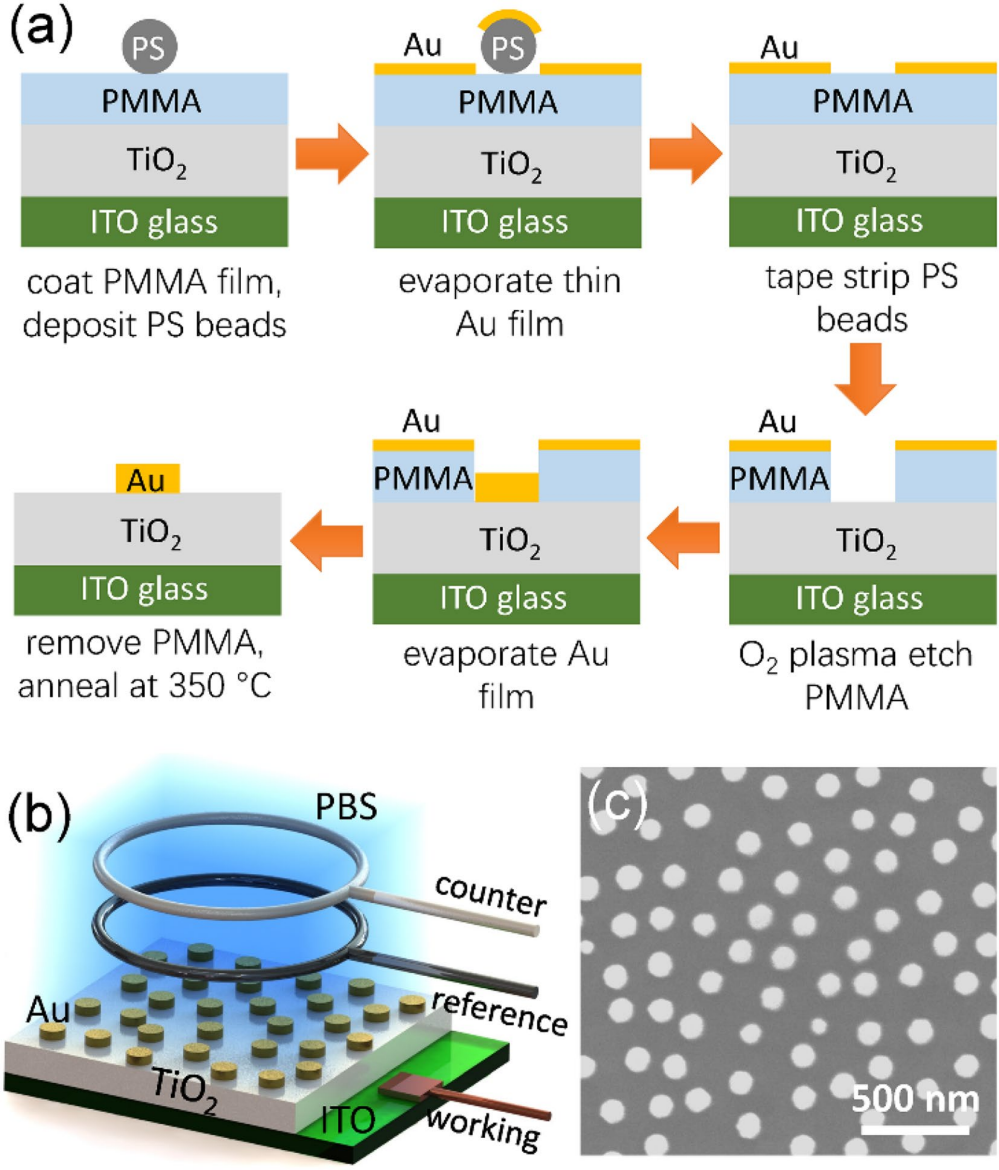
(**a**) Schematics of the HCL method for Au nanodisk fabrication. PMMA and PS are the abbreviations of polymethyl methacrylate and polystyrene, respectively. (**b**) Schematic structures of AT sample and electrochemical cell. (**c**) SEM image of AT sample with 100 nm nanodisk diameter.

AT structure was fabricated on ITO glass substrate. First, a 2 nm thick Cr adhesion layer was deposited on the substrate, followed by sputtering a 100 nm thick TiO_2_ film in an O_2_ (3 sccm) and Ar (50 sccm) plasma at 5 mTorr and 0.8 kW DC. Then, Au nanodisks with average diameter 80 nm or 100 nm, and height 30 nm were fabricated on the TiO_2_ film using hole-mask colloid lithography (HCL, Fig. 5a) followed by annealing at 350 °C. The structure and SEM image of AT sample are shown in Fig. 5b,c, respectively. Au nanodisks randomly distributed on TiO_2_ film, and their diameter is relatively uniform. In-Ga/TiO_2_/In-Ga and Au/TiO_2_/In-Ga structures were fabricated by coating In-Ga alloy layer, TiO_2_ film, and In-Ga alloy or Au layer in sequence. In-Ga/TiO_2_/ITO structure was also fabricated by sequentially coating TiO_2_ film and In-Ga alloy layer using ITO glass as substrate. Further details of sample fabrication are referred to the literature^37^.

### Photocurrent measurement

Photocurrents of AT structure were measured in a three-electrode system using an Ag/AgCl wire reference electrode, a Pt counter electrode, and standard phosphate buffer saline (PBS) electrolyte (Fig. 5b). Photocurrents were collected at chronoamperometric mode with constant bias voltages against reference electrode. Incident light with different wavelength was filtered out by an acousto-optic tunable filter (AOTF) from a laser driven light source (LDLS, Energetiq). Further detailed description of procedures for photocurrent measurements is referred to the literature^37^.
